# Disruption of cyanobacterial γ-aminobutyric acid shunt pathway reduces metabolites levels in tricarboxylic acid cycle, but enhances pyruvate and poly(3-hydroxybutyrate) accumulation

**DOI:** 10.1038/s41598-019-44729-8

**Published:** 2019-06-03

**Authors:** Tanakarn Monshupanee, Chayanee Chairattanawat, Aran Incharoensakdi

**Affiliations:** Department of Biochemistry, Faculty of Science, Chulalongkorn University, Bangkok, 10330 Thailand

**Keywords:** Applied microbiology, Metabolic engineering

## Abstract

The photoautotrophic cyanobacterium *Synechocystis* sp. PCC 6803 assimilates carbon dioxide as the sole carbon source, and a major portion of the assimilated carbon is metabolically consumed by the tricarboxylic acid (TCA) cycle. Effects of partial interference of TCA cycle metabolic activity on other carbon metabolism have yet to be examined. Here, the γ-aminobutyric acid (GABA) shunt, one of the metabolic pathways for completing TCA cycle in *Synechocystis*, was disrupted via inactivating the glutamate decarboxylase gene (*gdc*). Under normal photoautotrophic condition, cell growth and the level of the TCA cycle metabolites succinate, malate and citrate were decreased by 25%, 35%, 19% and 28%, respectively, in Δ*gdc* mutant relative to those in the wild type (WT). The cellular levels of glycogen and total lipids of the Δ*gdc* mutant were comparable to those of the WT, but the intracellular levels of pyruvate and bioplastic poly(3-hydroxybutyrate) (PHB) were 1.23- and 2.50-fold higher, respectively, in Δ*gdc* mutant. Thus, disruption of the GABA shunt pathway reduced the TCA cycle metabolites levels, but positively enhanced the bioaccumulation of pyruvate and PHB. The PHB production rate in Δ*gdc* mutant was 2.0-fold higher than in the WT under normal photoautotrophy.

## Introduction

The well-studied photoautotrophic cyanobacterium *Synechocystis* sp. PCC 6803 (hereafter, *Synechocystis)* assimilates carbon dioxide (CO_2_) as the sole carbon source. Two thirds (66% mole) of the assimilated carbon is metabolically consumed by glycolysis and the tricarboxylic acid (TCA) cycle, while the rest (34% mole) of the fixed carbon fluxes to other metabolism, including biosynthesis of the carbon reserve products: glycogen, total lipids and poly(3-hydroxybutyrate) (PHB)^1^. These carbon reserve products are of interest since glycogen and lipids, particularly fatty acids and hydrocarbons, are efficient substrates for the production of bioethanol^2,3^ and biodiesel^4–6^, respectively, while PHB can be utilized as a biodegradable plastic^7–9^. Thus, metabolic inhibition targeted on the TCA cycle would reduce the TCA cycle metabolic consumption, and so potentially could increase the cellular metabolic carbon flux towards other biochemical pathways, such as biosynthesis of the carbon reserve products. However, inhibition of the TCA cycle metabolic activity reduced the cell growth of *Synechocystis*^10^. Thus, a partial inhibition of the TCA cycle that does not cause deleterious effects to cell growth, but is capable of redirecting the carbon metabolic flux towards carbon reserve biosynthesis is a desirable strategy for the bioproduction of carbon reserve products.

The *Synechocystis* TCA cycle is distinct from the traditional TCA cycle in that it lacks the two steps for the conversion of 2-oxoglutarate to succinyl-CoA and then to succinate^10–12^. Recent experiments have shown that *Synechocystis* completes the TCA cycle using the (i) Oxo/Sucsem/Suc metabolic route (converting 2-oxoglutarate to succinyl semialdehyde and then succinate) and (ii) γ-aminobutyric acid (GABA) shunt metabolic route (converting 2-oxoglutarate to glutamate, GABA, succinyl semialdehyde and then succinate)^10,13^. Thus, disrupting one of these two routes may partially reduce the TCA cycle metabolic activity in *Synechocystis*.

Here, the *Synechocystis* GABA shunt route was disrupted with the aim to negatively interfere with the TCA-cycle metabolic activity via inactivating the glutamate decarboxylase (the enzyme in GABA shunt route for converting glutamate to GABA) gene (*gdc*). We asked whether *gdc* inactivation affects the (i) TCA cycle metabolic activity and (ii) has effects on the nearby metabolic pathways (PHB synthesis and glycolysis), and the more distal carbon reserve metabolisms of lipid and glycogen synthesis. We found that *gdc* inactivation reduced the metabolites levels in the TCA cycle, but increased the accumulation levels of PHB and pyruvate. Since, *gdc* inactivation has also been shown to significantly increase the glutamate (Glu) level in *Synechocystis*^14^, we next asked whether the increased PHB level mediated by *gdc* inactivation was a consequence from the increased Glu level or from the affected TCA cycle. To address this, a section of the metabolic network that links Glu metabolism, the TCA cycle and PHB synthesis was examined by analyzing relevant metabolites and enzymatic activities. In addition, the effects of an external Glu supply on the PHB level were investigated.

## Materials and Methods

### Strains and culture conditions

Wild type (WT) *Synechocystis* sp. PCC 6803 was obtained from the Pasteur culture collection. The Δ*gdc* mutant was constructed previously^14^ by deleting the *gdc* gene (*sll1641*) in the WT *Synechocystis*. Approximately 5% (v/v) of a 14-d-old cell culture was inoculated into BG-11 medium^15^ without citrate but supplemented with 20 mM HEPES-NaOH (pH 7.5). Cells were supplied by atmospheric CO_2_ and cultured at 30 °C under continuous white light at 50 µmol/m^2^/s. Culture in nitrogen-deprived (-N) or phosphorus-deprived (-P) condition was performed as previously described^16^. Sodium glutamate was supplied as Glu source when required.

### Analysis of PHB, glycogen and total lipid levels

Approximately 10 mg dry cells were used for the analyses, where PHB levels were determined as previously described^17^. Essentially, dry cells were boiled in 18 M sulphuric acid to convert PHB into crotonic acid, which was then quantified by HPLC analysis. Commercial PHB was examined in parallel, where a PHB conversion level to crotonic acid was obtained at 82 ± 5% (w/w). Glycogen was purified, enzymatically digested to glucose and the glucose levels were quantified using the glucose oxidase assay (GLUCOSE Liquicolor Kit, Human Gesellschaft fur Biochemica und DiagnosticambH, Germany) as previously described^16^. Total lipids were extracted and quantified using the acidic dichromate methods as previously described^16^.

### Analysis of metabolites

From a total of 250 mL of homogeneously-suspended cell culture, 50 mL was used for the dry cell mass determination and 200 mL was used for wet cell harvest. The organic acid metabolites (pyruvate, 2-oxoglutarate, succinate, malate and citrate) in the wet cells were extracted and quantified essentially as described before^18^, but with a slight modification in the HPLC analysis section (see below). Wet cells were frozen in liquid nitrogen, resuspended in methanol, sonicated and centrifuged. The supernatant was freeze-dried and reconstituted in methanol. Then, the organic acid metabolites were quantified using a Shimadzu (Japan) HPLC system according to the manufacturer’s protocol. The HPLC was equipped with an InertSustain Carbon-18 column and eluted in a 1:49 (v/v) of methanol: 0.1% (v/v) H_3_PO_4_ mobile phase with UV detection at 210 nm. Adipic acid was used as the HPLC internal standard.

Cellular glutamate (Glu) contents were quantified as previously described^19^. In brief, Glu was extracted from wet cells and subsequently derivatized with o-phthaldehyde and analyzed by HPLC using 338-nm detection. Acetyl-CoA levels were determined using the acetyl-CoA assay kit MAK039 (Sigma-Aldrich, MO, USA), where the acetyl-CoA is extracted and specifically catabolized by a coupled-enzymatic assay which resulted in a product that was quantified by its fluorescent intensity (535-nm excitation and 587-nm emission). Cellular NADPH levels were determined using an NADPH quantification kit MAK038 (Sigma-Aldrich, MO). Briefly, total NADP^+^ and NADPH were extracted from wet cells. Samples were then heated at 60 °C to decompose NADP^+^ and the obtained NADPH samples were quantified using a colorimetric assay, determined by the absorbance at 450 nm.

### Analysis of enzymatic activities

The PHA synthase activity was determined as described before^20^. The reaction was performed in Tris-glycerol buffer (5% v/v glycerol in 25 mM Tris-HCl pH 7.5) that contained 0.5 mM 5,5-dithiobis(2-nitrobenzoic acid), 1.5 mM 3-hydroxybutyryl-CoA and 0.2 mg/mL crude cell protein extract. The assay was incubated at 30 °C for 10 min and the thiobenzoate anion product was quantified spectrophotometrically at 412 nm. The crude protein concentration was determined using the Lowry method.

The NADP-dependent glutamate dehydrogenase activity was determined as described before^21^. The reaction was performed in 50 mM Tris-HCl pH 8.0 buffer containing 50 mM Glu, 2 mM NADP^+^ and 0.2 mg/mL crude protein extract. The assay was incubated at 30 °C for 10 min, and the product was determined by the absorbance at 340 nm.

### PHB extraction, NMR and material property analyses

The PHB was purified from dry cells as previously described^22^ and then analyzed by ^1^Hydrogen- and ^13^Carbon-nuclear magnetic resonance (NMR) at 25 °C using a Bruker Advance 400 MHz spectrometer (Germany). Physical properties were examined using a material analyzing machine (Hounsfield H10KM, UK). Thermal properties and polymer molecular weight was determined as described^23^.

## Results and Discussion

### Inactivation of *gdc* increased the PHB level under photoautotrophy

The GABA shunt metabolic route of *Synechocystis* was disrupted by inactivating the *gdc* gene encoding glutamate decarboxylase, to yield the Δ*gdc* mutant of *Synechocystis*, as previously reported^14^. Here, the effect of *gdc* inactivation on PHB accumulation was examined.

**Figure 1:**
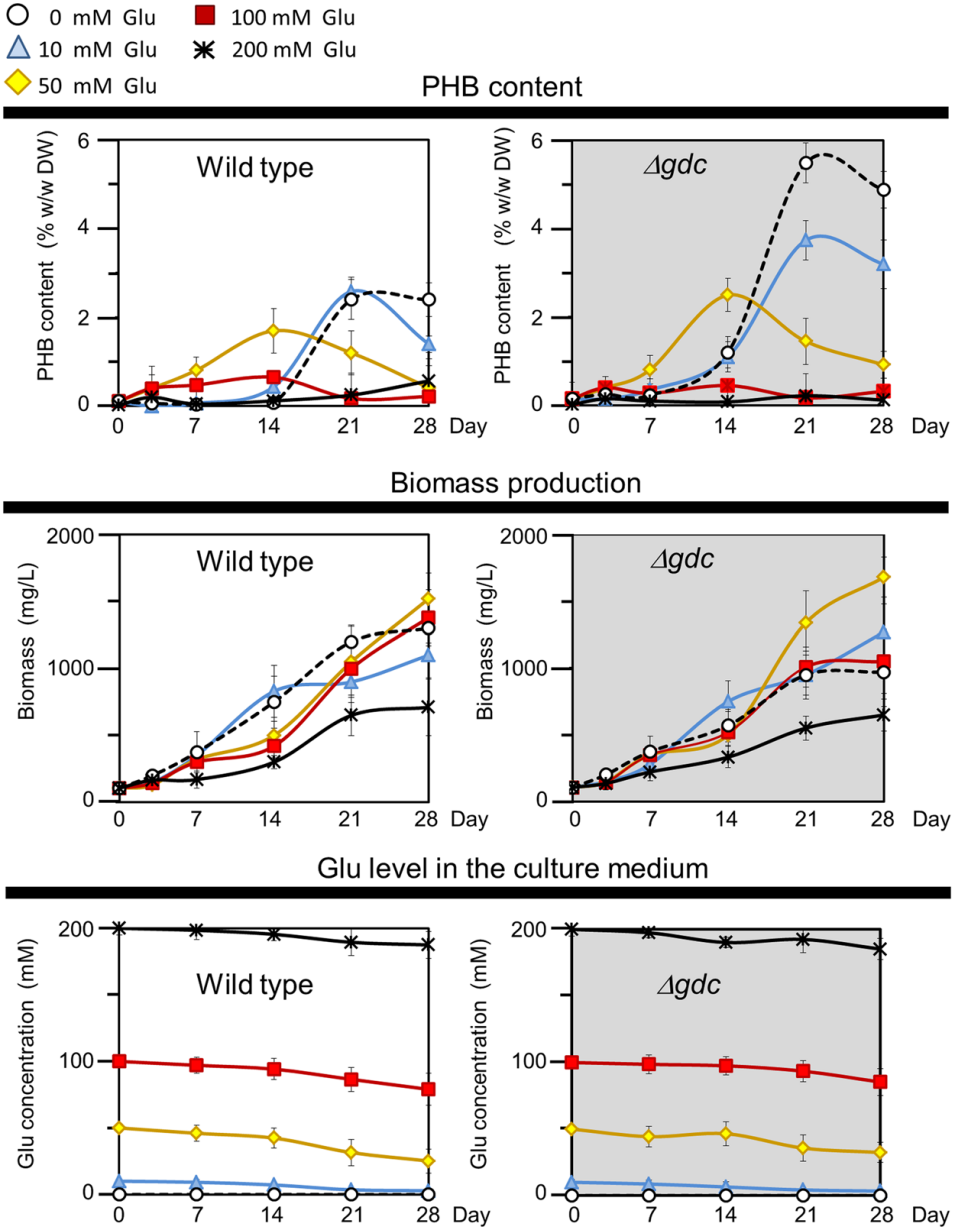
PHB accumulation and biomass levels under normal photoautotrophic condition. Log-growth-phase cells of the wild type (WT) and Δ*gdc* mutant were diluted and grown in the presence or absence of glutamate (Glu) at 0–200 mM. Data are shown as the mean ± 1 SD derived from three to six independent cultures.

Under the normal photoautotrophic condition (0 mM Glu supply), the wild type (WT) *Synechocystis* had a maximal PHB level of only 2.2% w/w dry weight (DW) at late-growth phase (after 21 d of culture), whereas the Δ*gdc* mutant had a 2.5-fold higher PHB level at 5.5% w/w DW (Fig. 1). However, a 1.25-fold higher biomass level was found in the WT than in the Δ*gdc* mutant (Fig. 1). The PHB accumulations were significantly increased in both wild type and Δ*gdc* mutant after 14 days of cultivation (Fig. 1). These increased PHB levels might be a response to the stress due to the depletion of phosphate and nitrate in the culture medium. The depletion of phosphate and nitrate after 10 and 15 d respectively was reported in *Synechocystis* cell culture using 250 ml of BG11 medium^24^, which are the same culture volume and medium used in the present study.

**Figure 2:**
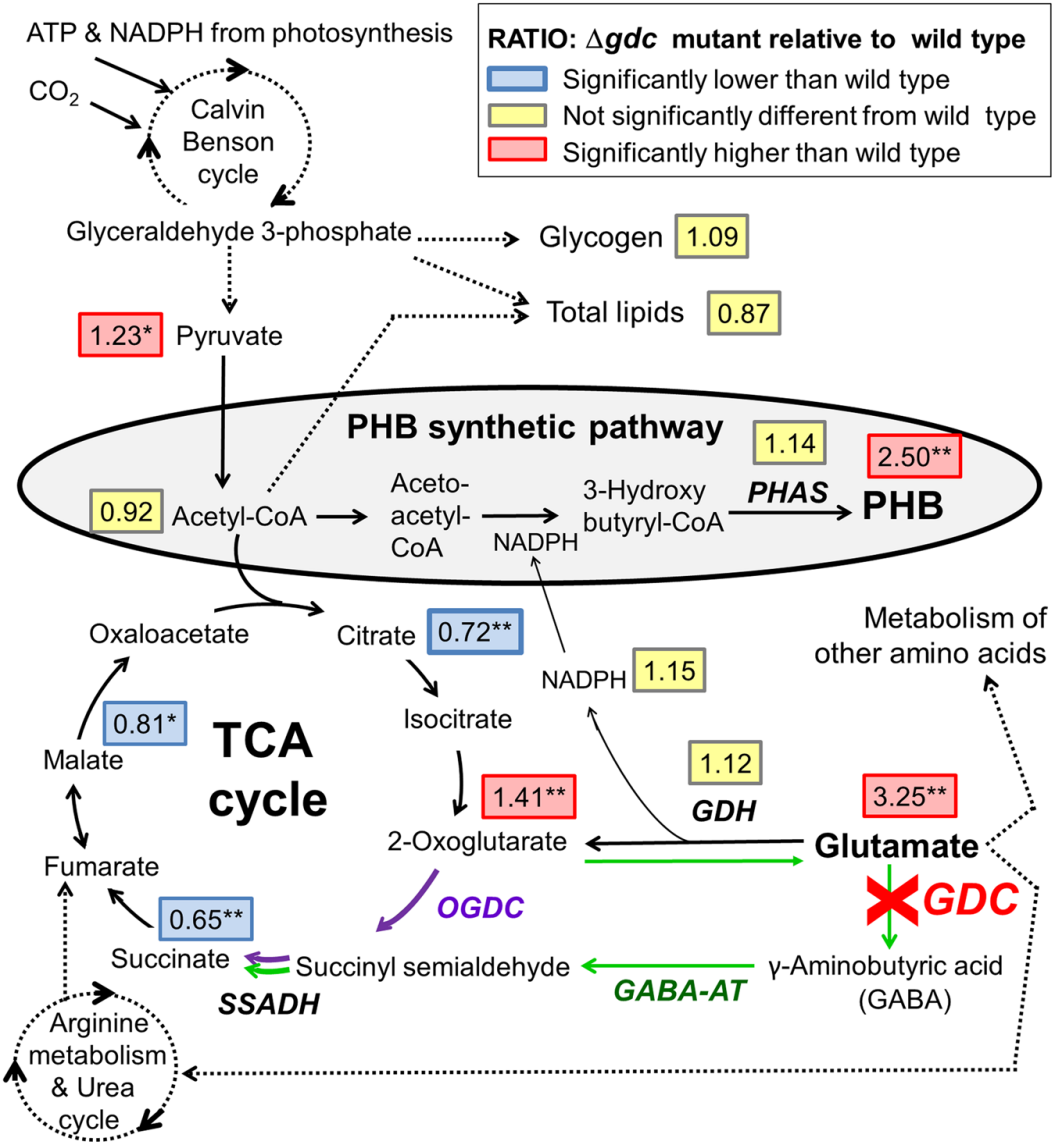
Metabolites levels and enzymatic activities of the Δ*gdc* mutant as relative values to those of the wild type (shown in rectangle boxes). Log-growth-phase cells were diluted and cultured under the normal photoautotrophic condition (0 mM Glu) for 21 d. Metabolites, direct reactions (line arrows), abbreviated pathways (broken arrows) and enzymes (italic capital letters: *GABA-AT*, γ-aminobutyrate aminotransferase; *GDC*, glutamate decarboxylase; *GDH*, glutamate dehydrogenase; *OGDC*, 2-oxoglutarate decarboxylase; *PHAS*, polyhydroxyalkanoate (PHA) synthase; *SSADH*, succinic semialdehyde dehydrogenase) are illustrated. The 2-oxoglutarate/succinyl semialdehyde/succinate route (Oxo/Sucsem/Suc route, shown by purple arrows) and GABA shunt route (green arrows) for completing the *Synechocystis* TCA cycle are indicated. Data are obtained from four to six independent cultures. Asterisks indicate the ratios for which the levels in Δ*gdc* were significantly different from those in the WT (*P < 0.05; **P < 0.01: unpaired two-tailed *t*-test). This metabolic connection section was redrawn from the reported metabolic network of *Synechocystis* sp. PCC6803^10–12,29^. The values of metabolites levels and enzymatic activities of the wild type (WT) and Δ*gdc* mutant are given in Table S1, Supplementary Information.

Since the Δ*gdc* mutant was found to have a higher intracellular level of glutamate (Glu) than the WT (Fig. 2), consistent with a previous report^14^, we examined whether this increased Glu level led to the elevated PHB accumulation in the Δ*gdc* mutant. External Glu was supplied to cell cultures with the aim of increasing the cellular Glu level, and then the PHB accumulation was quantified. Culture time periods of 21 and 28 d were chosen because these time periods showed the most apparent enhancement of PHB accumulation in the Δ*gdc* mutant (Fig. 1). However, no enhanced PHB levels in either the WT or Δ*gdc* mutant was evident after 21 and 28 d of culture at all supplied extracellular Glu levels (Fig. 1). Thus, the increased intracellular Glu level in the Δ*gdc* mutant cultured without Glu supply (Fig. 2) was unlikely to cause an increase of PHB level in the Δ*gdc* mutant (Fig. 1). In addition, Glu is possibly imported into the cells, since the supplied Glu concentrations in the culture medium were continuously declined throughout cell growth (Fig. 1). It has been also shown that *Synechocystis* can uptake Glu into the cells by ABC-type protein transporter^25^.

It is of note that the external Glu enhanced PHB accumulation at 50 and 100 mM Glu supply in the WT and at 50 mM Glu supply in the Δ*gdc* mutant during the early growth phase (d7 and d14) (Fig. 1). This external Glu-mediated PHB accumulation is unlikely a result from the ability of the cells to use Glu as carbon source because no significantly increased biomass level was detected under Glu supplementation. The externally supplied Glu may contribute to the increase of intracellular level of glutamate which may be subsequently metabolized by glutamate dehydrogenase to regenerate NADPH required for PHB synthesis.

### *gdc* inactivation did not increase PHB accumulation under nitrogen-deprived (-N) or phosphorus-deprived (-P) condition

**Figure 3:**
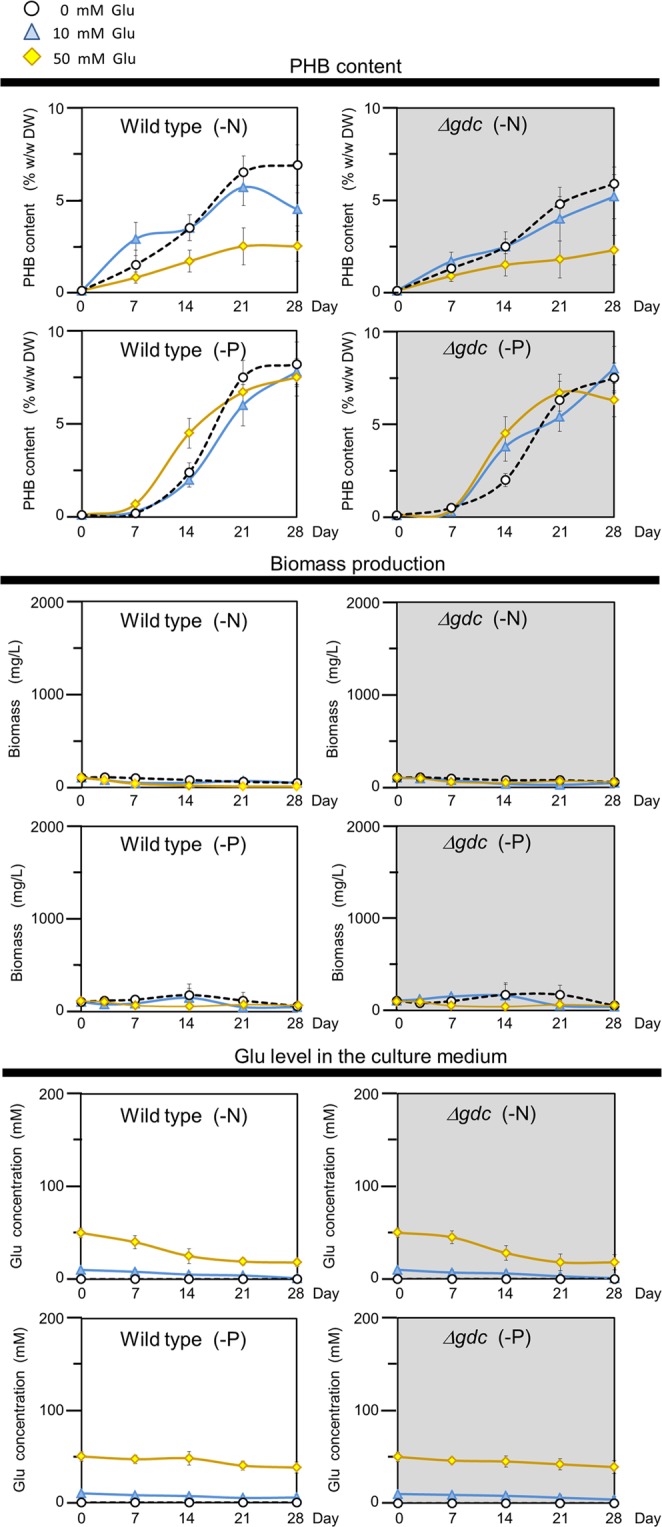
PHB and biomass levels under nitrogen deprivation (-N) or phosphorus deprivation (-P). Log-growth-phase cells of the wild type (WT) and Δ*gdc* mutant were diluted and cultured in either -N or -P medium in the presence of glutamate (Glu) at 0, 10 or 50 mM. Data are shown as the mean ± 1 SD derived from three or four independent cultures.

Culturing of *Synechocystis* under -N or -P condition has been shown to significantly enhance the level of PHB accumulation^16,26,27^. Accordingly, we determined the effect of a -N or -P condition on the PHB accumulation in the Δ*gdc* mutant and WT *Synechocystis*. Regardless of the presence or absence of an external Glu supply, the Δ*gdc* mutant and WT had comparable PHB levels under either a -N or a -P condition (Fig. 3). Thus, inactivation of *gdc* had no apparent effect on PHB accumulation in *Synechocystis* under a -N or -P condition. In addition, no cell growth was observed under either a -N or -P condition in both the *gdc* mutant and WT cells (Fig. 3).

### Effects of *gdc* inactivation on the TCA cycle and related metabolism

Here we explored the metabolic changes responsible for the increased PHB level in the Δ*gdc* mutant compared to the WT. The metabolic linkage section that connects the GABA shunt metabolic route, TCA cycle, PHB biosynthesis and Glu metabolism (Fig. 2) was analyzed for their respective metabolites levels and enzymatic activities.

With respect to Glu metabolism, the Δ*gdc* mutant had a 3.25-fold increased cellular Glu level relative to the WT (Fig. 2), which was likely due to the lack of glutamate decarboxylase (GDC), the enzyme that catalyzes the conversion of Glu to GABA. Since there is no reported metabolic pathway for converting Glu to PHB, the increased Glu level in the Δ*gdc* mutant seems unlikely to be the cause of the increased PHB accumulation. The cellular activity of glutamate dehydrogenase (GDH), which converts Glu to 2-oxoglutarate and releases NADPH (the reducing co-factor for PHB synthesis) was found to be comparable in the Δ*gdc* mutant and the WT cells (Fig. 2). Thus, the GDH activity is not the contributing factor for the increased PHB accumulation of the Δ*gdc* mutant.

With respect to PHB synthesis, the levels of acetyl-CoA (the primary substrate for PHB synthesis), NADPH (the reducing co-factor for PHB synthesis) and cellular polyhydroxyalkanoate synthase activity (the last enzyme in PHB synthetic pathway) of the Δ*gdc* mutant and WT were at comparable levels (Fig. 2), suggesting that all these three factors are not likely to be the principal causes for the elevated PHB storage in the Δ*gdc* mutant.

With respect to the TCA cycle, *gdc* inactivation directly disrupted the GABA shunt, one of the main metabolic routes for completing the TCA cycle in *Synechocystis*^10^. In the present study, *gdc* inactivation increased the level of 2-oxoglutarate (an upstream TCA cycle metabolite) by 1.41-fold, but significantly decreased the levels of the subsequent TCA cycle metabolites, i.e., succinate, malate and citrate by 0.35-, 0.19- and 0.28-fold, respectively (Fig. 2). Thus, the GABA-shunt disruption negatively interfered with the TCA cycle metabolic activity, particularly at the steps that generate succinate, malate and citrate from 2-oxoglutarate, as evident by the high 2-oxoglutarate level, but the low succinate, malate and citrate levels. The 25% reduction of the specific growth rate seen in the Δ*gdc* mutant (Table 1) supports this contention of the negative effect of *gdc* inactivation on TCA cycle metabolites levels. However, it should be noted that the HPLC analysis system used in this study exhibited a low UV absorption signal towards other TCA cycle metabolites (oxaloacetate, isocitrate and fumarate, data not shown), thus making it difficult to accurately determine these metabolites levels by this technique.

**Table 1:** PHB production and growth rate under different culture conditions and glutamate (Glu) supplies in the wild type (WT) and Δ*gdc* mutant.

Nutrient condition	Glu supply (mM)	Maximum specific PHB production rate (mg PHB/g CDW/d)		Specific growth rate at exponential growth phase (d^−1^)	
		WT	Δ*gdc*	WT	Δ*gdc*
Normal	0	3.0 ± 0.7	**6.1 **± 0.7**	0.36 ± 0.03	0.27 ± 0.03**
	10	3.0 ± 0.3	**3.8 **± 0.3*	0.35 ± 0.05	0.23 ± 0.04**
	50	0.1 ± 0.0	0.1 ± 0.0	0.34 ± 0.04	0.25 ± 0.03**
Nitrogen deprivation	0	4.3 ± 0.5	3.3 ± 0.5	NA	NA
	10	3.1 ± 0.3	**2.1 **± 0.2**	NA	NA
	50	1.1 ± 0.1	**0.0 **± 0.0**	NA	NA
Phosphorus deprivation	0	7.3 ± 0.9	6.1 ± 1.0	NA	NA
	10	5.7 ± 0.6	**2.3 **± 0.8**	NA	NA
	50	3.1 ± 0.3	3.1 ± 0.2	NA	NA

Approximately 5% (v/v) of log-growth-phase cells were inoculated and cultured. The maximum specific PHB production rates were obtained during the culture period from d 14 to 21. The specific growth rates were calculated based on cell dry weight during 7 d period of exponential growth. Data are the average ± 1 SD of three to five cultures. Asterisks indicate a significantly different value (*P < 0.05; **P < 0.01: two-tailed *t-*test) than that acquired from the WT under the same nutrient condition and Glu supply. NA, not applicable, since no cell growth was detected.

The TCA cycle and PHB synthesis have a direct metabolic linkage where both are competing for the same primary substrate - acetyl-CoA (Fig. 2). We speculated that the negative interference on the TCA cycle with the declined metabolites level seen in the Δ*gdc* mutant (Fig. 2) might reduce the acetyl-CoA consumption by the TCA cycle. This would potentially lead to acetyl-CoA molecules being available to flux towards PHB biosynthesis. However, as the steady-state level of acetyl-CoA in the Δ*gdc* mutant and the WT cells were comparable (Fig. 2), therefore the elevated PHB level in the Δ*gdc* mutant was not likely to result from an increased steady-state acetyl-CoA level, but would rather be a consequence of the increased flux rate of acetyl-CoA towards PHB synthesis. Further metabolic flux analysis that measures the rate of acetyl-CoA consumption by the TCA cycle and by the PHB synthetic pathway in the Δ*gdc* mutant is required to provide an insight into the cellular mechanism responsible for the increased PHB level in the mutant.

With respect to lipid metabolism, *gdc* inactivation did not significantly alter the total lipid content (Fig. 2), suggesting that *gdc* inactivation has no general effect on lipid metabolism. *Synechocystis* total lipids are mainly composed of diacylglycerol membrane lipids^28^, which are produced from a complex biosynthesis network^11,29^ and fall within the limited range of 10–16% w/w DW under normal photoautotrophy^16,30^.

### Effects of interruption of GABA shunt on carbon reserve levels

Photoautotrophic *Synechocystis* assimilates CO_2_ as the sole carbon source. Recent ^13^CO_2_-isotopic flux analysis showed that 66% of the fixed carbon was directed to glycolysis and the TCA cycle, 10% to glycogen synthesis and 24% to various other metabolic pathways, including the biosynthesis of total lipids and PHB^1^. Here, we found that the reduced TCA-cycle metabolites levels in the Δ*gdc* mutant had a positive effect on the nearby metabolic pathways, including a 1.23- and 2.5-fold increased level of pyruvate (downstream glycolytic metabolite) and PHB, respectively (Fig. 2). However, no apparent effect on the more distal bioproduction of total lipids and glycogen was observed (Fig. 2).

In *Synechocystis*, two main metabolic routes for completing the TCA cycle have been experimentally confirmed; namely the (i) Oxo/Sucsem/Suc route (converting 2-oxoglutarate to succinyl semialdehyde and then succinate) and (ii) GABA shunt route^10^ (Fig. 2). However, a third potential route for completing the TCA cycle is via arginine metabolism (Fig. 2), although this has not yet been confirmed^10^. The disruption of the Oxo/Sucsem/Suc route in *Synechocystis* has been shown to interfere with the TCA cycle leading to a 0.43- to 0.68-fold decreased succinate level and a 0.25-fold declined cell growth^10^. Similarly, disrupting the Oxo/Sucsem/Suc route in another cyanobacterium (*Synechcoccus* sp. PCC 7002) reduced the levels of succinate and malate in the TCA cycle by 0.8- and 0.5-fold, respectively, but increased 2-oxoglutarate accumulation by 2.3-fold^31^. Likewise, disrupting the *Synechocystis* GABA shunt route (via deleting the gene encoding GABA aminotransferase, see Fig. 2) reduced the succinate level 0.78-fold and decreased cell growth by 0.20-fold^10^. Nevertheless, the effects on other metabolisms have yet to be examined. Here, we found that interrupting the *Synechocystis* GABA shunt (via Δ*gdc*) also reduced the TCA cycle metabolites succinate, malate and citrate, but increased the bioaccumulation of PHB and pyruvate (Fig. 2).

Further intensifying the inhibitory effect on the TCA cycle, particularly at metabolic rate-limiting steps, might help redirect more of the acetyl-CoA pool towards PHB synthesis. Since the interference with the TCA cycle found in the Δ*gdc* mutant also affected the cell growth (Fig. 1 and Table 1), a programmed switchable culture capable of turning on the TCA cycle for optimal cell growth, and then turning it off for PHB production would effectively improve the PHB production level by cyanobacteria. This switchable TCA cycle approach might be done via a conditionally-inducible gene expression system, as recently described^32,33^.

### PHB production by the Δ*gdc* mutant

The normal photoautotrophic condition is known to yield a high biomass production but low PHB accumulation in *Synechocystis*^16,26,27^. Enhancing PHB accumulation in *Synechocystis* has been achieved by culturing cells under a -N or -P condition; however, under either condition the biomass production is extremely reduced^16,26,27^, which was also the case in this study (Figs 1 and 3). To obtain a high biomass production and PHB accumulation in cyanobacteria, a two-stage culture has been established, where cells were first pre-grown under normal photoautotrophy to acquire a high biomass yield, and then subsequently subjected to the -N or -P condition to obtain a high PHB level^7–9,23^. Nevertheless, this two-stage culture requires a prolonged time and consumes more energy, which restricts its production feasibility.

Therefore, a single-stage culture approach capable of simultaneously yielding a high cell biomass and high PHB levels would be advantageous. Such a strategy was primarily demonstrated in this study, where the Δ*gdc* mutant was cultured under a single-stage normal photoautotrophy to produce a relatively high biomass yield (0.98 g/L or 75% of the WT level) and an elevated PHB accumulation of 5.5% w/w DW, a 2.5-fold higher than that of the WT (Fig. 1). Overall, the Δ*gdc* mutant has the maximum specific PHB production rate at 6.1 mg PHB/g CDW/d, a 2.0-fold greater than that obtained from the WT (Table 1) under this single-stage culture approach. Higher PHB production and productivities have been achieved in randomly UV-mutated *Synechocystis* sp. PCC 6714^34^. A number of metabolic engineering approaches for increasing *Synechocystis* PHB accumulation were recently reviewed^35,36^. Further combining such metabolic engineering strategies together with the *gdc* inactivation as described in this study would likely help enhance PHB production by *Synechocystis*.

### Material properties of the PHB obtained from the Δ*gdc* mutant

The purified PHB from Δ*gdc* mutant showed ^13^C- and ^1^H-NMR spectra that matched the respective NMR spectra from the commercial PHB (Supplementary Data Fig. S1). The thermal and mechanical properties of PHB from the Δ*gdc* mutant were comparable to those of the commercial PHB (Table 2), except for a 15% lower Young’s modulus of elongation found in the Δ*gdc*-mutant PHB. It is noted that the PHB obtained from the Δ*gdc* mutant had a 30–40% lighter molecular weight (*M*_*w*_ and *M*_*n*_) and a 14% lower polydispersity relative to the commercial PHB (Table 2).

**Table 2:** Material properties of the PHB obtained from the *Synechocystis* Δ*gdc* mutant.

Source of PHB	Thermal property					Mechanical property^d^			Molecular weight		
	*T*_*m*_ (°C)	*T*_*g*_ (°C)	*T*_*cc*_ (°C)	*∆H*_*m*_ (J/g)	*X*_*c*_ (%)	Elongation at break (%)	Tensile strength (MPa)	Young’s modulus (MPa)	*M*_*w*_ (kDa)	*M*_*n*_ (kDa)	*M*_*w*_/*M*_*n*_
Commercial PHB^a^	175.4 (159)	3.5	48	99	68	5.8 ± 1.1	24 ± 3	820 ± 300	970	330	2.9
*Calothrix scytonemicola* ^b^	170.7 (164)	4.1	47	88	60	4.2 ± 1.6	38 ± 7	724 ± 284	415	196	2.1
*Synechocystis Δgdc* ^c^	170.4 (163)	6.7	53	90	62	3.8 ± 1.9	**28 **±** 7**	**701 **±** 255**	**586**	**234**	**2.5**

*T*_*m*_, melting temperature (first melting peak shown in parentheses); *T*_*g*_, glass-transition temperature; *T*_*cc*_, cold-crystallization temperature; *∆H*_*m*_, enthalpy of fusion; *X*_*c*_, % crystallinity; *Mw*, weight-average molecular weight; *Mn*, number-average molecular weight; *Mw/Mn*, polydispersity.

^a^Heterotrophic bacterial PHB. Data are from Sigma-Aldrich (USA).

^b^Photoautotrophic cyanobacterial PHB^8^.

^c^Photoautotrophic cyanobacterial PHB. Cells were cultured under normal growth condition for 21 d (this study).

^d^Mechanical properties are the mean ± 1 SD of three independent experiments.

## Conclusions

Disruption of the GABA shunt via *gdc* inactivation slightly affected the cell growth, but significantly increased the pyruvate and PHB levels in *Synechocystis*. The results suggested that this increased PHB level might be due to the negative interference on the TCA cycle metabolism, which may then allow more acetyl-CoA molecules to flux towards PHB synthesis. Further intensifying the inhibitory effect on TCA cycle metabolic function, together with inactivation of metabolic pathways that largely consume the cellular metabolic carbon pool, such as glycogen synthesis, may redirect more metabolic carbon flux towards the biosynthesis of carbon reserve products (such as PHB) in *Synechocystis*.

## Supplementary information

Disruption of cyanobacterial γ-aminobutyric acid shunt pathway reduces metabolites levels in tricarboxylic acid cycle, but enhances pyruvate and poly(3-hydroxybutyrate) accumulation
