# Supplementary material for: Disruption of cyanobacterial γ-aminobutyric acid shunt pathway reduces metabolites levels in tricarboxylic acid cycle, but enhances pyruvate and poly(3-hydroxybutyrate) accumulation
Source: Sci Rep. 2019 Jun 3;9:8184. doi: 10.1038/s41598-019-44729-8 (PMC6547876; doi:10.1038/s41598-019-44729-8)

Supplementary Information

Disruption of cyanobacterial γ-aminobutyric acid shunt pathway reduces metabolites levels in tricarboxylic acid cycle, but enhances pyruvate and poly(3-hydroxybutyrate) accumulation

Tanakarn Monshupanee*, Chayanee Chairattanawat and Aran Incharoensakdi

Department of Biochemistry, Faculty of Science, Chulalongkorn University, Bangkok 10330, Thailand. **Correspondence:* T.M.; E-mail*:* tanakarn.m@chula.ac.th

Table S1. Intracellular levels of metabolites and cellular enzymatic activities of the wild type and Δ*gdc* mutant of *Synechocystis* sp. PCC 6803.

Cells were cultured as described in Fig. 2. Data are shown as the average ± 1SD of four to six independent cultures. Asterisks indicate significantly different values from the WT (*, $P < 0.05$; **, $P < 0.01$: unpaired two-tailed *t*-test). Red and blue highlight show significantly higher and lower, respectively, levels in *Δgdc* than in the WT. Note: glycogen, total lipids and PHB have a wide range of molecular masses, hence their contents cannot be calculated to mol/g DW. Thus, the contents of such three groups of compounds were determined as in % (w/w DW).

**Metabolite content**			
**Metabolite**	**Content unit**	**WT**	***Δgdc* mutant**
2-Oxoglutarate	µmol/g DW	0.12 ± 0.02	**0.17 ± 0.02****
Acetyl-CoA	µmol/g DW	0.66 ± 0.11	0.61 ± 0.13
Citrate	µmol/g DW	0.56 ± 0.06	**0.40 ± 0.05****
Glutamate	µmol/g DW	12.12 ± 1.23	**39.39 ± 5.59****
Glycogen	% w/w DW	10.03 ± 2.58	10.93 ± 2.70
Malate	µmol/g DW	0.18 ± 0.01	0.15 ± 0.01*
NADPH	nmol/g DW	1.43 ± 0.45	1.64 ± 0.42
Total lipids	% w/w DW	15.32 ± 3.45	13.33 ± 4.12
PHB	% w/w DW	2.20 ± 0.50	**5.49 ± 0.45****
Pyruvate	µmol/g DW	0.75 ± 0.05	**0.92 ± 0.10***
Succinate	µmol/g DW	0.15 ± 0.02	**0.10 ± 0.01****
**Enzymatic activity**			
**Enzymes**	**Activity unit**	**Wild type**	***Δgdc* mutant**
Polyhydroxyalkanoate synthase (PHA synthase)	nmol/min/mg protein	4.65 ± 0.75	5.30 ± 0.72
Glutamate dehydrogenase	nmol/min/mg protein	10.69 ± 2.52	11.97 ± 3.10

Fig. S1. ^{1}Hydrogen-NMR (upper) and ^{13}Carbon-NMR (lower) spectra of the PHB obtained from the *Δgad Synechocystis* sp. PCC6803. Cells were grown as mentioned in Fig. 2. The NMR peaks corresponding to each hydrogen and carbon atom in the PHB chemical structure are indicated by numerical numbers.

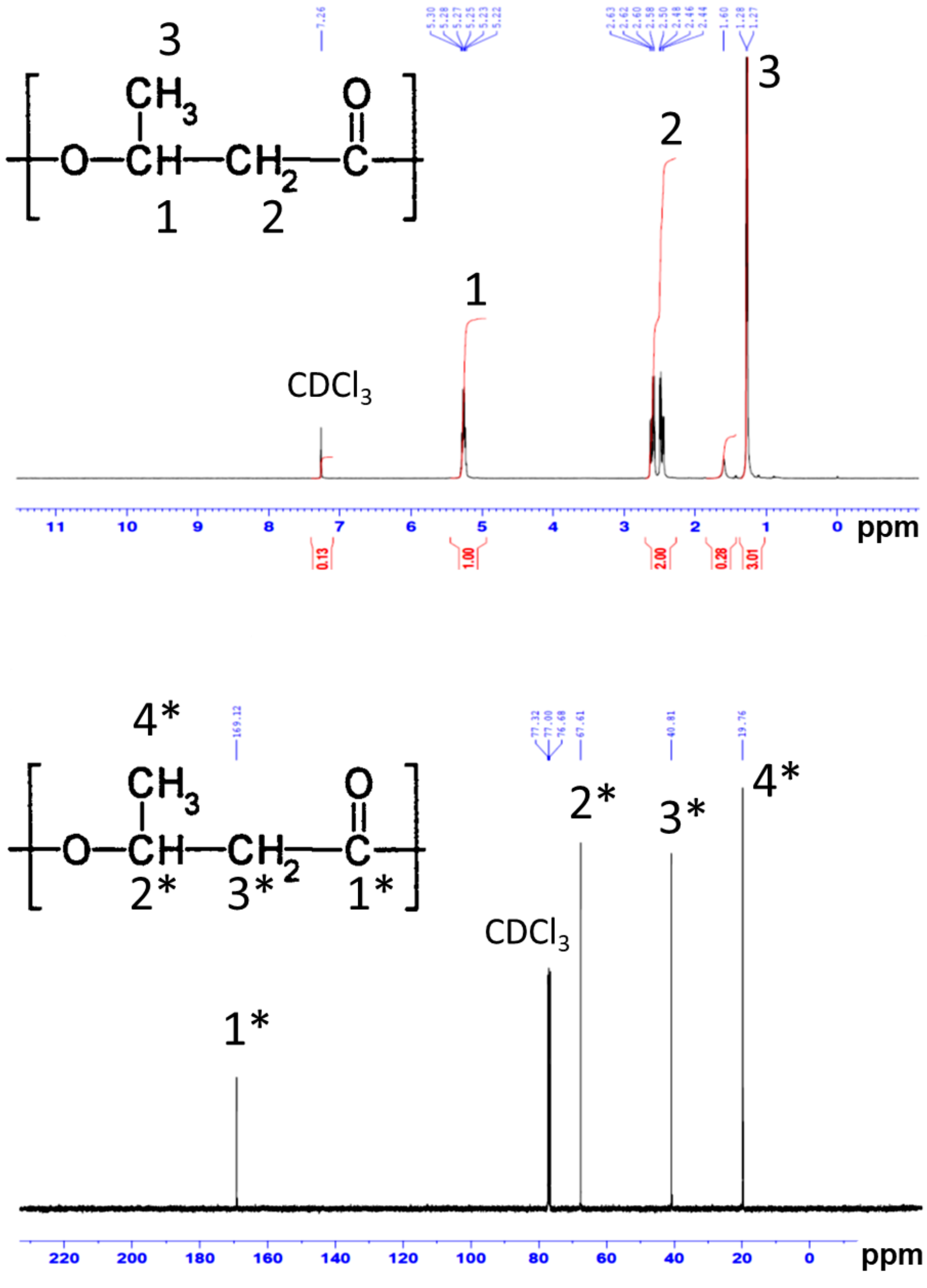

Supplement: Supplementary file 1 — Disruption of cyanobacterial γ-aminobutyric acid shunt pathway reduces metabolites levels in tricarboxylic acid cycle, but enhances pyruvate and poly(3-hydroxybutyrate) accumulation [file 41598_2019_44729_MOESM1_ESM.pdf]
